# Significantly enhanced electrocatalytic activity of copper for hydrogen evolution reaction through femtosecond laser blackening

**DOI:** 10.1016/j.ijhydene.2020.12.174

**Published:** 2021-03-11

**Authors:** Zihao Li, Sohail A. Jalil, Subhash C. Singh, Weishan Li, Xiaoming Wei, Chunlei Guo

**Affiliations:** a School of Physics and Optoelectronics, South China University of Technology, Guangzhou 510640, China; b The Institute of Optics, University of Rochester, Rochester, NY 14627, United States; c School of Chemistry, South China Normal University, Guangzhou 510631, China

**Keywords:** Femtosecond laser, Black copper, Electrocatalyst, Hydrogen evolution reaction

## Abstract

In this work, we report on the creation of a black copper via femtosecond laser processing and its application as a novel electrode material. We show that the black copper exhibits an excellent electrocatalytic activity for hydrogen evolution reaction (HER) in alkaline solution. The laser processing results in a unique microstructure: microparticles covered by finer nanoparticles on top. Electrochemical measurements demonstrate that the kinetics of the HER is significantly accelerated after bare copper is treated and turned black. At −0.325 V (v.s. RHE) in 1 M KOH aqueous solution, the calculated area-specific charge transfer resistance of the electrode decreases sharply from 159 Ω cm^2^ for the untreated copper to 1 Ω cm^2^ for the black copper. The electrochemical surface area of the black copper is measured to be only 2.4 times that of the untreated copper and therefore, the significantly enhanced electrocatalytic activity of the black copper for HER is mostly a result of its unique microstructure that favors the formation and enrichment of protons on the surface of copper. This work provides a new strategy for developing high-efficient electrodes for hydrogen generation.

## Highlights

• Black Cu is fabricated via fs-laser processing as a novel electrode for HER.
• Fs-laser processing yields a unique surface of Cu with micro/nano-particles.
• The resultant electrode exhibits a significantly accelerated HER kinetics.

## Graphical abstract

**Figure:**
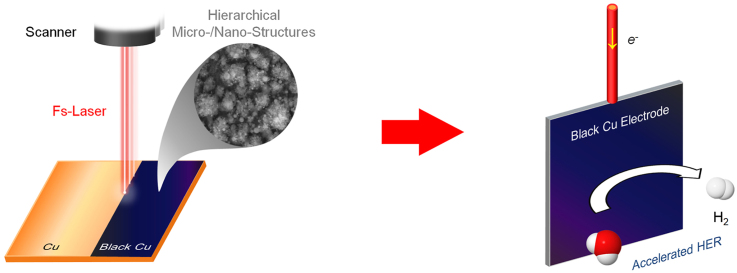

## Introduction

After the concept of hydrogen economy was brought up in the 1970s [1], the hydrogen industry blooms across the world with a rapidly growing need of hydrogen [2]. Over large scale, commercially mature production of hydrogen has been made possible through both thermochemical conversion and electrolysis methods. Thermochemical conversion (including gasification, steam reforming and partial oxidation) emits byproducts and requires feedstock of carbon-based fuels such as coal, alcohol, hydrocarbon, carbon monoxide, or biomass, which are not comparable to water, the feedstock of electrolysis [3]. Hydrogen generation by electrolyzing water and its application as fuel in fuel cells are most sustainable when electricity is produced from solar or wind energy, compared with other electrochemical energy conversion and storage systems [[4], [5], [6], [7], [8], [9]].

Platinum (Pt) and platinoids such as iridium (Ir), palladium (Pd) and rhodium (Rh) are active electrocatalysts for hydrogen evolution reaction (HER) [10], but their use is mostly an issue because of abundance [11]. Various electrocatalysts have been developed for replacing Pt and platinoids [[12], [13], [14]]. Cu is one of the most promising substitutes, because the abundance of Cu in the Earth's crust is four orders of magnitude more than Pt [15], and Cu has a more positive standard electrode potential than hydrogen, which makes it stable in non-oxidative aqueous solutions [16,17]. The activity of electrocatalysts on anodes or cathodes for any electrochemical energy conversion devices is highly related to their compositions and microstructures [[18], [19], [20]], especially for HER [21]. Cu has been investigated in various compositions and microstructures as electrocatalysts for HER. The most focused form is the complex of Cu, whose electrocatalytic activity is originated from the redox process in the complex [22]. Several composites of Cu with other elements or compounds have also been fabricated, such as Cu–Ni alloy [23], Cu–Ni_3_S_2_ composite [24], Cu_2_Se [25], and Cu–C composite [26]. These investigations are undoubtedly helpful for the application of Cu as cathodes for hydrogen generation from water electrolysis. However, comparatively, less attention has been paid to the contribution of Cu surface microstructure to its electrocatalytic activity towards HER.

Laser processing is a viable technique for the creation of various microstructures to alter the optical [27], mechanical [28], and electrical [29] properties of materials such as aluminum [30], silicon [31], titanium [32], and gold [33] for a wide variety of applications in photonics, plasmonic and energy fields [34]. Compared to chemical etching and plasma ablating, femtosecond (fs) laser processing has higher accuracy, efficiency, controllability as well as repeatability, and can introduce microstructures on materials with higher physical stability. The creation of periodic microstructures and porous features showed significant enhancement in the field emission properties [35]. Fs-laser processed metal electrodes have already found useful in electrocatalyzing hydrogen [36] and oxygen [37] evolution reactions. Fs-laser processed Cu electrode was applied as support of catalyst [38], but few reports are available on the applications of fs-laser processed Cu as electrocatalysts. Encouragingly, nanospikes on Cu have been found to be beneficial for electrochemical reduction of aqueous CO_2_ into formaldehyde [39]. This phenomenon is attributed to the accumulation of free electron on the spikes that create a strong electric field and enrich the reactant species, leading to the enhanced electrocatalytic activity. Here, for the first time, we report the highly enhanced electrocatalytic activity of Cu towards HER with a unique surface microstructure induced by fs-laser. We show that fs-laser processing turns bare Cu surface to a black surface that provides Cu electrode with significantly enhanced HER kinetics.

## Experimental section

The black Cu, Cu foil treated by fs-laser, was fabricated through scanning a fs-laser beam on an 1 mm thick Cu foil (named as bare Cu electrode) using SCANcube III 10 galvanometer scanner (SCANLAB GmbH, Germany) equipped with 10 cm focal length f-q lens. The surface microstructures of the treated Cu depend on the power and the scanning speed of the laser beam. The average power of the laser beam was 1.8 W. The laser beam was scanned on the Cu foil with an scanning speed of 1 mm s^−1^ and an interline spacing of 0.15 mm. The fs-laser pulse was generated by Asterella-USP-1K ultrafast Ti:sapphire amplifier system (Coherent Inc., USA) with wavelength centered at 800 nm, pulse width of ~35 fs, pulse repetition frequency of 1 kHz, and beam quality M^2^ less than 1.25. The backside of every sample was soldered to a electrical wire and black Cu sample was placed in a way that its grooves introduced by fs-laser ablation were perpendicular to the horizontal line.

Scanning electron microscopy (SEM) image of black Cu surface was taken using JSM-6480 LV scanning electron microscope (JEOL Ltd., Japan) to observe the fs-laser-induced surface microstructure of Cu. For electrochemical measurements, a three-electrode electrolytic cell was used, with black Cu or bare Cu as working electrode, Ag/AgCl in 3.5 M KCl solution as reference electrode and Ti as counter electrode. The potentials were reported with respect to standard hydrogen electrode (RHE). The electrolyte used was 1 M NaOH aqueous solution. Linear sweep voltammetry (LSV) and electrochemical impedance spectroscopy (EIS) were performed on VSP-300 electrochemical workstation (BioLogic Company, Inc., France). The sweep rate for LSV was 20 mV s^−1^. The potential for CA and EIS was −0.325 V. The alternating current signals for EIS measurement was in the frequencies ranging from 100 kHz to 100 mHz with a voltage amplitude of 5 mV. The working electrodes were tailored to the same size of 1 × 1 cm^2^ and their backside was sealed using silicone. All the electrochemical measurements were done at room temperature (20 °C). The impedance simulation was performed using software Zview (Scribner Associates Inc., USA).

## Results and discussion

**Fig. 1:**
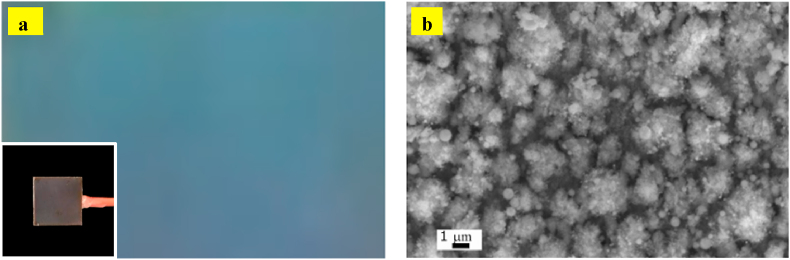
Close-in photograph for femtosecond laser-treated Cu surface with an inset showing the whole electrode (a) and SEM image of the femtosecond laser-induced surface microstructure on black Cu (b).

Fs-laser processing can strongly modify properties of the treated surfaces. Fig. 1a shows a photographic image of the fs-laser-treated Cu foil. By mere inspection, we can see that after fs-laser processing, the surface of bare Cu is darkened and presents a blueish colour. The fs-processed Cu foil is named as black Cu (here after). Fig. 1b is an SEM image of the surface microstructure of black Cu obtained by raster-scanning of the laser beam on the sample surface. The formation of the structural colour is attributed to the excitation localized plasmon resonance, and the spectral shift is due to plasmonic hybridization [40,41]. As a result of Cu ablation under laser irradiation and redeposition of nanoparticles, a unique microstructure (microparticles covered by finer nanoparticles) is formed, which provides sharp tips that would converge the electric field when electrode is polarized, and, in turn, enhance the local field intensity.

For an HER in the alkaline solution, the abundant OH^−^ ions do not directly participate in HER [42]. Instead, the HER at electrode/electrolyte interface involves the formation of H^+^ ion (proton) from water dissociation (Eq. (1)), the adsorption of generated protons on the electrochemical active sites of the electrode (Volmer step, Eq. (2)) and the combination of the two adsorbed hydrogen atoms to form hydrogen gas (Tafel step, Eq. (3)) [43],

(1) $$2H_{2}O\;\to \;2H^{+}\;+\;2OH^{-}$$

(2) $$H^{+}\;+\;e^{-}\;\to \;H_{ad}$$

(3) $$2H_{ad}\;\to \;H_{2}$$

Accordingly, an electrode that possesses high electrocatalytic activity towards HER should favor the formation of proton, the adsorption and the combination of hydrogen atoms. The black Cu electrode under negative polarization is favorable to these processes. The resulting strong electric field is beneficial for the formation of free protons from water dissociation and the adsorption of proton through electric-field induced water dissociation and the adsorption of generated protons on the electrode-electrolyte interface. On the other hand, the microstructure with rough and irregular-shaped nanoparticles on the black Cu electrode provides more suitable sites for the combination of the adsorbed hydrogen atoms than the flat surface on the bare Cu electrode. Furthermore, the vertically structured grooves of black Cu also provide pathways for the evolution of hydrogen bubbles generated during electrocatalysis and thus can improve the detached behavior of hydrogen. Therefore, an enhanced electrocatalystic activity of the black Cu electrode towards HER can be expected

**Fig. 2:**
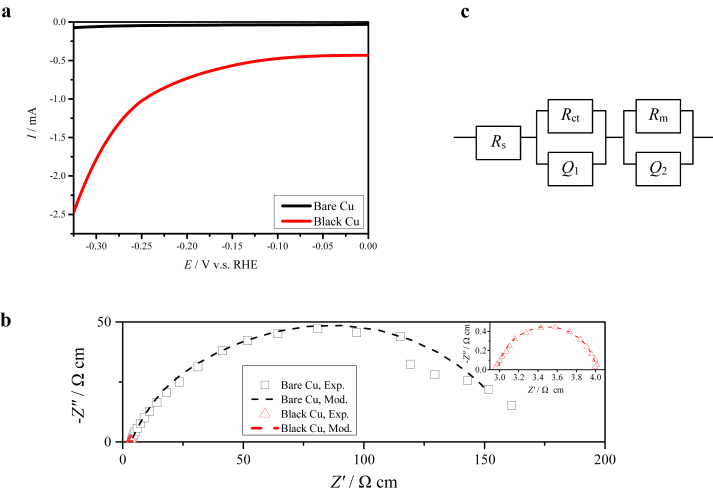
Linear sweep voltammograms (a), electrochemical impedance spectra at −0.325 V (b) and equivalent circuit for impedance simulation (c) for bare Cu and black Cu electrodes in 1 M NaOH solution.

Fig. 2a presents the linear sweep voltammograms of the bare and the black Cu electrodes in 1 M NaOH solution. Small potential-dependent current density can be observed on the bare Cu electrode. Besides the minor oxygen dissolved in the solution, whose reduction is controlled by diffusion process and independent of potential, there are no other species that can be reduced on the electrode in NaOH solution. Therefore, the recorded potential-dependent current represents the HER. The small current densities suggest that the bare Cu electrode exhibits low electrocatalytic activity toward HER. As the potential shifts negatively, the current density increases, indicative of the charge-transfer controlled HER, but the current increase is insignificant. Even at −0.3 V, the current density on the bare Cu electrode merely reaches −0.06 mA cm^−2^. By contrast, fast increase in the current density can be observed on the black Cu electrode as the potential swifts negatively. At −0.3 V, a current density of −1.78 mA cm^−2^ is harvested on the black Cu electrode, which is 30 times more than that on the bare Cu electrode, indicating that the black Cu electrode exhibits excellent electrocatalytic activity towards HER. It is obvious that the electrocatalytic activity of Cu towards HER can be highly enhanced by the fs-laser treatment. The enhanced electrocatalytic activity towards HER results from the accelerated kinetics of HER (Eqs.(1), (2), (3)) on the black Cu electrode with the unique surface microstructure induced by fs-laser, which can be further confirmed by electrochemical impedance spectroscopy.

Fig. 2b presents the electrochemical impedance spectra of the bare Cu and black Cu electrodes at −0.325 V in 1 M NaOH solution. The experimental data can be well fitted with the equivalent circuit shown in Fig. 2c, which includes *R*_s_, *R*_ct_//*Q*_1_ and *R*_m_//*Q*_2_, representing solution resistance, the characteristics of interface for charge transfer and the unevenness of the micro-/nano-structures on electrode surfaces, respectively [44]. The obtained *R*_s_ from modeling is 3 Ω cm^2^ for both electrodes, indicative of the same contribution of the solution to the impedance. The obtained area-specific surface resistance *R*_m_ is 10 Ω cm^2^ for the bare Cu electrode, which is far larger than that for the black Cu electrode (2 × 10^−4^ Ω cm^2^), suggesting that the surface microstructure of the black Cu is more compatible with the solution than that of the bare Cu, which is beneficial for overcoming water dissociation energy barrier [45]. Compared with the small *R*_s_ and *R*_m_, the bare Cu electrode presents a large area-specific charge transfer resistance (*R*_ct_ = 159 Ω cm^2^), confirming the slow kinetics of HER on the bare Cu electrode. In contrast, the black Cu electrode has a small *R*_ct_ (1 Ω cm^2^), which is even smaller than *R*_s_ (3 Ω cm^2^), suggesting that the HER on the black Cu electrode is hardly dependent of the charge transfer process. These impedance comparisons apparently confirm that the electrocatalytic activity of Cu towards HER can be highly enhanced by the surface microstructure introduced by fs-laser.

**Fig. 3:**
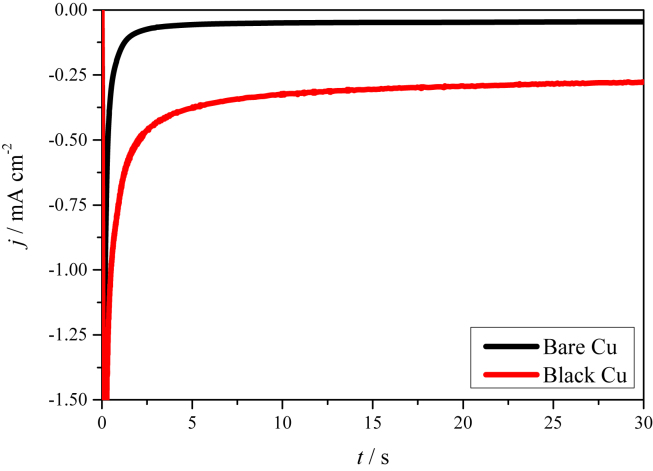
Chronoamperograms of bare Cu and black Cu electrodes at −0.325 V in 1 M NaOH solution.

There is the posibility that nanoparticles might be aggregated and the hierarchical structure of the black Cu electrode might consequently be damaged when HER proceeds in the corrosive solution. To identify the stability of the surface structure induced by fs-laser, chronoamperometry was performed on black Cu electrode at −0.325 V, with a comparison of bare Cu electrode. As shown in Fig. 3, the black Cu electrode presents a far larger current density than the bare Cu electrode, indicative of the highly enhanced electrocatalytic activity of black Cu electrode towards HER. Most importantly, the recorded current does not show significant decrease at the end of chronoamperometry, suggesting that the hierarchical micro- and nano-structure introduced by fs-laser is stable, which is important for the practical application of the black Cu electrode.

**Fig. 4:**
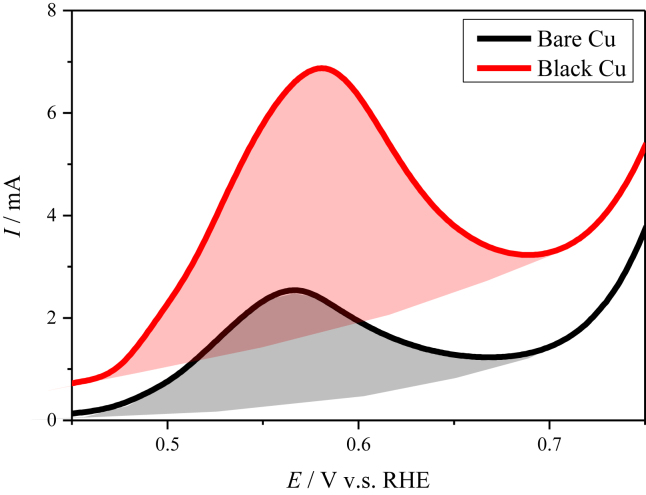
Linear sweep voltammograms for bare Cu and black Cu in 1 M NaOH solution.

It should be noted that the surface microstructure of the black Cu electrode provides large electrochemical surface area (ECSA) that might contribute to larger current densities than the bare Cu electrode. Usually, ECSA is calculated by scanning the CV curves at different scanning speeds in the non-faraday regions to obtain double layer capacitance (C_dl_). To be more directly, the ECSA of both electrodes was compared with their linear sweep voltammograms. As shown in Fig. 4, a characteristic peak can be found around −0.4 V in forward sweeping of Cu electrodes, which reflects the oxidation of surface Cu atoms and represents the available Cu surface. Therefore, the ECSA of Cu can be estimated by integrating the curves at the potentials between 0.45 V and −0.7 V, as indicated by the shaded area in Fig. 4. The resulting ratio of the ECSA between the black Cu and the bare Cu electrodes is 2.4:1. This ratio is far smaller than those of the current density from linear sweep voltammograms (Fig. 2a) or the reciprocal *R*_ct_ (Fig. 2b), confirming that the highly enhanced electrocatalytic activity of the black Cu electrode results from its unique microstructure, while less dependent of its increased ECSA. This unique microstructure yields strong electric field under negative polarization, which favors the formation of free protons from water dissociation, the adsorption of proton, and the combination of the adsorbed hydrogen atoms, leading to the significantly accelerated HER kinetics.

## Conclusion

Femtosecond (fs) laser can be used to develop unique surface microstructures on copper, which exhibit a highly enhanced electrocatalytic activity toward hydrogen evolution reaction in alkaline solution. These microstructures feature with rough and irregular-shaped nanoparticles, which can create strong local electric field when the Cu electrode is under negative polarization. The resulting strong electric field favors the formation of free protons from water dissociation, the adsorption of proton, and the combination of the adsorbed hydrogen atoms, leading to the significantly accelerated HER kinetics. As a result, the Cu electrode after fs-laser treatment presents a 30 times’ enhancement of the hydrogen evolution current. This is the first time to report the application of fs-laser in the fabrication of Cu electrode for electrochemical generation of hydrogen. The electrocatalytic activity of the fabricated Cu electrode could be further improved by delicately designing the surface microstructures with changing the fabrication conditions such as the power and the scanning speed of fs-laser.

## Declaration of competing interest

The authors declare that they have no known competing financial interests or personal relationships that could have appeared to influence the work reported in this paper.
